# Supplementary material for: Using mobile audiometry (Wulira App) to assess noise induced hearing loss among industrial workers in Kampala, Uganda: A cross-sectional study
Source: PLoS One. 2023 Jan 6;18(1):e0279407. doi: 10.1371/journal.pone.0279407 (PMC9821776; doi:10.1371/journal.pone.0279407)
Supplement: Attachment — Submitted filename: Response to reviewers.docx [file pone.0279407.s001.docx]

**Reviewer #1: It is a very good study, but I have a doubt. The industry had 900 employees, 300 were excluded for not working exposed to noise. That gives me 600 and not 400 employees exposed to noise. Is it a mistake?**

Response: Thank you for your comment. Yes, this is mistake in the write up. The actual number exposed to noise is 400 as outlined in the study population section of the manuscript. The actual number of employees excluded is 500. The tables have been updated as such.

**Reviewer #2: There is a difference in the rate of hearing loss between ears. Was this significant? The authors include this fact in the discussion, nevertheless, this an unexpected finding in workers exposed to noise coming from several sources simultaneously. There was a difference between low-frequency and high-frequency hearing loss, the former being more prevalent. This is also an unexpected finding since occupational hearing loss is characterized by high-frequency hearing loss.
The last two comments lead me to check the original publication about the Wulira app. The paper previously published by the authors showed that the app had a good sensitivity and specificity to detect subjects with normal hearing. But the sensitivity dropped to approximately 53% when subjects had mild hearing loss, and even below 50% for more severe cases. Maybe the app should be employed as a screening tool for identifying subjects with normal hearing levels, but the use as a diagnostic method of levels of hearing loss is still questionable. This issue could explain the findings of differences between ears and the higher prevalence of low-frequency hearing loss.
This is the reason I do not recommend this paper for publication**

Response: Thank you for your comment. Yes, there is a statistically significant difference between the hearing loss between the ears. The P value is less than 0.001.

It is true that this is an unexpected finding among workers exposed to noise from different directions/sources simultaneously. Previous research has, however, noted differences in hearing loss by ear side among Finnish individuals exposed to chronic noise (1) while other researchers have demonstrated asymmetry in OHL (2).This has been added to the discussion lines 253—259.

Regarding the high prevalence of low frequency noise induced hearing loss, this contrasts with previous studies as we have highlighted in our discussion previously. We also agree that the frequency of “high frequency hearing loss” should be more for noise induced hearing loss than that seen in our study. The other possible explanation for this could be the inherent Wulira app performance as you highlight. This has been added to the discussion, line 270—271. The other underlying diseases/conditions for causing low frequency hearing loss like Meniere’s disease in our population were not ruled and this has been added as a limitation, line 303—304.

We have also highlighted throughout the manuscript that the tool should be used as a screening tool.

**Reviewer #3: The paper study the prevalence of occupational hearing loss among workers of metal manufacturing company located in Kampala, Uganda. The assessment was performed using the Wulira App. It was found that about 10 percent of the workers of this company had occupational hearing loss. The results highlight the importance of hearing loss screening among this populations and the usefulness of the Wulira App as an alternative to pure tone audiometry as a screening tool.

The paper is well written and clear. The methodology is sound, and the results are interesting. For these reasons, I recommend accepting the paper after the following issues are addressed:**

*** Major Issues**

**- Show some examples of the audiometry obtained with the Wulira App. For instance, show one audiometry of a subject with occupational hearing loss and one of a subject without it. This will allow the reader to get a better understanding of the kind hearing loss observed in the population studied.**

Response: Thank you for your comment. These have been added

**- Use a bar plot for figure 2. See** [**https://en.wikipedia.org/wiki/Pie_chart#Use_and_effectiveness**](https://en.wikipedia.org/wiki/Pie_chart#Use_and_effectiveness) **for the rationale about avoiding pie charts. I suggest checking this reference (in particular section 6.1) for some guidelines about appropriately presenting bar graphs:** [**https://clauswilke.com/dataviz**](https://clauswilke.com/dataviz) **.**

Response: Thank you for your comment. A bar plot has been added

**- Line 202: Explain why reporting the religion is relevant. And if it is, please also report the other religions of the population.**

Response: Thank you for your comment. Religion is not important and hence has been removed.

**- In the paragraph starting at line 239 you compare the prevalence of your results with other studies and suggests some reasons to explain the differences. One factor that you do not consider explaining the difference is the use of the Wulira App. Is it possible that differences in the results are because you use a different tool to get the audiogram? Please comment about this issue in the discussion section.**

Response: Thank you for your comment. We included a sentence explaining the fact that other studies used PTA as compared to ours that utilized Wulira App. See lines 243—244.

*** Minor Issues
- Line 68: Change "Occupational hearing loss" by "Occupational Hearing Loss."**

Response: Thank you for your comment. This have been addressed.

**- Line 91: The sentence "Much as the Constitution ..." is hard to read. Please re-write to facilitate the understanding.**

Response: Thank you for your comment. This have been addressed.

**- Line 94: Add a coma before and after of "which is the gold standard for hearing loss screening."**

Response: Thank you for your comment. This have been addressed.

**- Line 97: Add a coma before and after "which is hearing loss screening using mobile phones and tablets."**

Response: Thank you for your comment. This have been addressed.

**- Line 197: Change "achieved. (Figure 1)" by "achieved (Figure 1)."**

Response: Thank you for your comment. This have been addressed.

**- Line 225: Change "respectively. (Table 4)" by "respectively (Table 4)."**

Response: Thank you for your comment. This have been addressed.

**References.**

1.	Pirilä T, Sorri M, Jounio-Ervasti K, Sipilä P, Karjalainen H. Hearing asymmetry among occupationally noise-exposed men and women under 60 years of age. Scandinavian Audiology. 1991;20(4):217-22.

2.	Le TN, Straatman LV, Lea J, Westerberg B. Current insights in noise-induced hearing loss: a literature review of the underlying mechanism, pathophysiology, asymmetry, and management options. Journal of Otolaryngology-Head & Neck Surgery. 2017;46(1):1-15.
